# Mobilization of CD34^+^-Progenitor Cells in Patients with Severe Trauma

**DOI:** 10.1371/journal.pone.0097369

**Published:** 2014-05-14

**Authors:** Ulrike Ritz, Volker Spies, Isabella Mehling, Dominik Gruszka, Pol Maria Rommens, Alexander Hofmann

**Affiliations:** BiomaTiCS-Group, University Medical Centre of the Johannes Gutenberg University, Center of Orthopaedic and Trauma Surgery, Mainz, Germany; French Blood Institute, France

## Abstract

Circulating CD34+ progenitor cells () gained importance in the field of regenerative medicine due to their potential to home in on injury sites and differentiate into cells of both endothelial and osteogenic lineages. In this study, we analyzed the mobilization kinetics and the numbers of CD34+, CD31+, CD45+, and CD133+ cells in twenty polytrauma patients (n = 13 male, n = 7 female, mean age 46.5±17.2 years, mean injury severity score (ISS) 35.8±12.5 points). In addition, the endothelial differentiation capacity of enriched CD34+cells was assessed by analyzing DiI-ac-LDL/lectin uptake, the expression of endothelial markers, and the morphological characteristics of these cells in Matrigel and spheroid cultures. We found that on days 1, 3, and 7 after a major trauma, the number of CD34+cells increased from 6- up to 12-fold (p<0.0001) over the number of CD34+cells from a control population of healthy, age-matched volunteers. The numbers of CD31+ cells were consistently higher on days 1 (1.4-fold, p<0.01) and 7 (1.3-fold, p<0.01), whereas the numbers of CD133+ cell did not change during the time course of investigation. Expression of endothelial marker molecules in CD34+cells was significantly induced in the polytrauma patients. In addition, we show that the CD34+ cell levels in severely injured patients were not correlated with clinical parameters, such as the ISS score, the acute physiology and chronic health evaluation II score (APACHE II), as well as the sequential organ failure assessment score (SOFA-2). Our results clearly indicate that pro-angiogenic cells are systemically mobilized after polytrauma and that their numbers are sufficient for the development of novel therapeutic models in regenerative medicine.

## Introduction

Musculoskeletal injuries are often accompanied by extensive vascular damage and local ischemia. In general, the processes of angiogenesis and *de novo* vasculogenesis represent key mechanisms of wound healing by restoring local blood supply and promoting tissue regeneration. Currently, it is generally understood that circulating stem and progenitor cells contribute to the repair of damaged tissues [1]. In particular, there is a wealth of evidence that circulating bone marrow-derived endothelial progenitor cells (EPCs) represent an important fraction of endothelial cells, as they have been shown to be involved in the repair and regeneration of blood vessels in animal models of ischemic tissue damage and myocardial infarction [2]–[4].

Circulating stem and progenitor cells have an innate ability to engage in vascular repair, but the mechanisms behind this are poorly defined. Endothelial progenitor cells were first discovered by Asahara *et al*. as CD34^+^ progenitor cells that derive from bone marrow [5]. CD34^+^ cells represent a heterogeneous population of cells with subpopulations that have different genetic and biological characteristics. These cells express a broad range of diverse surface markers and contain progenitor cells that are capable of differentiating into both endothelial and osteogenic lineages under the appropriate stimulating conditions [6]. However, it should be emphasized that the whole spectrum of bone-marrow derived CD34+ cells contains cell fractions that do not present functional characteristics of progenitor or of stem cells. Preclinical studies have shown that despite of their heterogeneity, human CD34+cells can stimulate neovascularization in ischemic myocardium by increasing capillary density and improving function in models of acute and chronic myocardial ischemia [7]. Clinical trials in the field of cardiovascular medicine also provided evidence that enriched pools of autologous CD34+ cells can improve clinical outcome results when administered by intramyocardial, intravascular, or intramuscular injection and supported further clinical development of this treatment strategy [8], [9].

Matsumoto *et al*. showed that CD34+cells are recruited to fracture sites and contribute to fracture healing when they are intravenously injected into nude rats with non-healing femoral fractures [6]. Although the mechanism of EPC mobilization from bone marrow is not fully understood, there is accumulating evidence that musculoskeletal trauma may cause a systemic, pro-vascular response in rodent bone marrow [10]–[12] and in human [13]–[15] bone marrow.

Although the number of publications on circulating progenitor cells has increased exponentially over the last few years, no data presently exist about the prevalence of these cells and their function in patients with multiple traumas. Patients who have been subjected to multiple traumas (or polytrauma) are described by a condition of multiple simoultanously occuring high-energy injuries of different body regions (for example serious head injury, blunt chest or abdomen trauma etc.) with one injury or the combination of injuries being dangerous to life. Despite of successful resuscitation, these patients are often at high risk to deteriorate unexpectedly due to “second hit” processes caused by severe tissue and organ damages. Therefore, this group of trauma patients in particular represents a potential candidate pool for therapeutic approaches in regenerative medicine.

The aim of this study was to analyze and specify mobilization kinetics, cell numbers, and the endothelial differentiation capacity of human CD34^+^ progenitor cells in severely injured patients. This study sheds new light on the question of whether CD34+cells are systemically activated in patients with severe trauma and are available in sufficient numbers for the development of new therapeutic models.

## Materials and Methods

### Patient collective

Patients enrolled in the study fulfilled the criteria of having polytrauma at admission according to definitions of Tscherne et al.[16], Trentz [17], and the German S3-guideline on treatment of patients with severe and multiple injuries [18] (severe trauma- (ST)-Group; Table 1).

**Table 1:** Polytrauma patients recruited to the study.

Internal patient ID	Age	Gender	ISS	Injury pattern
28	55	m	43	TBI, multiple maxillofacial fractures, BCT
29	51	m	34	open extremity injuries, severe skin degloving, BCT, TBI
30	62	m	18	multiple extremity injuries, BCT
33	22	m	33	TBI, BCT, multiple extremity fractures
34	20	m	42	BCT, BAI, SR, multiple extremity fractures
37	51	m	25	pelvic ring injury, multiple extremity fractures, BAI
46	27	m	34	BCT, pelvic ring fracture, extremity fractures
47	74	m	48	BAI, stomach and colon rupture, BCT, extremity fractures
52	50	m	29	BCT, multiple extremity open fractures, pelvic ring injury
53	22	m	45	BCT, BAI, pelvic ring injury, urinary bladder rupture, lumbosacral plexus injury
54	26	m	34	BCT, BAI, SR, multiple extremity fractures
56	62	m	20	pelvic ring injury, BAI, spine fractures, multiple extremity fractures
57	73	m	41	TBI, BCT, multiple extremity fractures
38	53	f	25	multiple open extremity injuries, spine injury, BAI
39	43	f	48	BAI, BCT, spleen and liver rupture, TBI, extremity fractures
40	67	f	41	TBI, BCT, multiple extremity fractures
45	31	f	75	TBI, BCT, pelvic ring fracture, multiple extremity fractures
55	54	f	36	BCT, pelvic ring fracture, extremity fractures
60	51	f	34	TBI, BAI, SR, spine fracture, extremity fractures
61	37	f	37	BCT, BAI, SR, LR, stomach rupture, pancreas rupture, multiple extremity fractures
		**Median**	**35 (IQR 13)**	**TBI: n = 7, BCT: n = 15, BAI: n = 10, Pelvic ring: n = 7**

TBI: traumatic brain injury; BCT: blunt chest trauma; BAI: blunt abdominal injuries; SR: spleen rupture; LR: liver rupture.

### Ethics statement

The ethical committee of the “Landesärztekammer Rheinland-Pfalz” approved the investigations (No.: 837.046.03(3708)), which conformed to the principles of the Helsinki Declaration. Informed consent was taken from each patient according to the approved protocol, or in case of unconsciousness or death, from their legal representatives. Initial resuscitation, diagnostic procedures, and primary surgical interventions were performed according to the guidelines of the German Society of Trauma Surgery. Patients were recruited at the intensive care unit. Blood samples were obtained from a consecutive series of 20 patients in a non-randomized prospective manner. Transfusions with leukocyte-depleted, packed red blood cells, fresh-frozen plasma, and thrombocyte concentrates were provided to patients in accordance with their standard of care.

### Quantification of circulating CD34^+^ progenitor cells in patients with severe trauma

Peripheral blood mononuclear cells (PBMCs) were isolated by density gradient centrifugation on days 1 (d1), 3 (d3), and 7 (d7). At each time point, samples of 20 ml of venous blood were collected in 7.5 ml S-Monovettes containing 1 ml CPDA (Sarstedt, Nümbrecht, Germany). Blood samples were diluted 1∶3 with phosphate buffered saline (PBS) containing 2 mM EDTA and separated on Ficoll-Paque (PAA Laboratories, Pasching, Austria) at 400×g and 20°C for 40 min. To remove thrombocyte residues, the pellet was subsequently washed two times in PBS/EDTA and once in PBS/EDTA/BSA. Cell counts were performed using the standard Trypan blue staining in a Neubauer hemocytometer.

Cell populations were quantified by fluorescence activated cell sorting (FACSCalibur, BD Biosciences, Heidelberg, Germany) using specific antibodies against the following cell surface markers: CD34 (FITC-mouse anti-human CD34 IgG2a, clone AC136, Miltenyi Biotec GmbH), CD133 (PE-mouse anti-human CD133 IgG1, clone AC133, Miltenyi Biotec GmbH), CD31 (APC-mouse anti-human CD31 (PECAM-1) IgG1, clone AC 128, Miltenyi Biotec GmbH), and CD45 (APC- or FITC-mouse anti-human CD45 IgG2a, clone 5B1, Miltenyi Biotec GmbH). Mouse anti-human IgG antibodies with conjugated dyes (clone IS5-21F5, Miltenyi Biotec GmbH) were used as negative isotype controls for all staining procedures. Control cultures were isolated from the peripheral venous blood of fourteen healthy volunteers and treated under the same conditions. To assure that no physiological fluctuations in the number of PBMCs, CD34+, CD31+, CD45+, CD133+, CD34+/CD45-, and CD31+/CD45- cells existed in the control group, FACS measurements were performed at three different time points (days 1, 3, and 7) according to the measurements in the ST-group. For statistical analysis, the number of positive cells was corrected for the total number of PBMCs.

### Isolation and culture of CD34^+^ progenitor cells

CD34^+^-cells were isolated from PBMC suspensions using magnetically labeled CD34-antibodies (QBEND/10, CD34 MicroBead Kit, MiniMACS-Separator, Miltenyi Biotec, Germany) according to the manufacturer's protocol. To ensure the purity of separated cells prior to the main experiments several pre-tests including FACS and immunofluorescence to ensure specificity of staining procedures and the correct binding of the antibody were performed. The pre-tests revealed median purity of 96% of CD34^+^ cells. CD34+cellswere counted, re-suspended in endothelial basal medium-2 (EBM-2 with supplement kit, 10% FCS; LONZA, Walkersville, USA; 100 U/ml penicillin, 100 µg/ml streptomycin sulphate) and cultured on fibronectin-coated 24-well plates at a densitiy of 100.000 cells/well in a humidified atmosphere (5% CO_2_, 37°C), with the media changed twice a week.

### Analysis of the endothelial markers in CD34^+^PC cultures

Formation of colony forming units (CFU) of elongated, spindle-like cells was routinely monitored in CD34+ cultures using a phase contrast microscope three times a week. The endothelial differentiation capacity of CD34+cells was verified in fifth-passage cultures using immunofluorescence staining for the following markers of mature endothelial cells: CD31 (PECAM-1, mouse anti-human CD31, clone Pecam1, DaKo; second antibody: anti-mouse IgG-Alexafluor546, Invitrogen), von Willebrand factor (vWF, mouse anti-human vWF, clone F8186, Dako, second antibody: anti-mouse IgG-FITC, Miltenyi), and CD146 (PE-mouse anti-human CD146 IgG1, Miltenyi Biotec, Bergisch Gladbach, Germany) [19], [20].

The phagocytosis of low density lipoprotein and the specific binding of Ulex europeus lectin to endothelial membrane receptors were investigated in purified CD34+ cell cultures by double staining with DiI-ac-LDL and lectin, as described elsewhere [21]. CD34+PC were purified as described above (ST-group: at days 1, 3, and 7 after severe trauma; control group: day 1). Cells were plated on fibronectin-coated 24-well *plates* and allowed to adhere for 24 hours prior to the staining procedure. The nuclei were stained with Hoechst 33258 (10 min, 4 g/ml; Sigma). The stained cells were visualized by fluorescence microscopy and quantified using ImageJ software (WSR, National Institutes of Health).

The vasculogenic potential was assessed by the analysis of primitive network formation on Matrigel and of endothelial sprout formation in cell cultures grown in carboxymethylcellulose (CMC) spheroids, as originally described and validated by Korff and Augustin [22], [23]. CD34+PC were purified as described above (ST-group: at days 1, 3, and 7 after severe trauma; control group: day 0) and subsequently cultured on fibronectin-coated 24-well plates. CD34-depleted cell fractions have been cultured under same conditions and used as negative controls. Sub-confluent cultures were trypsinized, washed, and resuspended in EBM-2 (Lonza) containing the supplement kit, 10% FCS and 0.25% (w/v) carboxymethylcellulose. Five hundred cells were pipetted into each well of non-adhesive round-bottom 96-well plates (Greiner, Germany). The spheroids were collected and suspended in an ice-cold collagen Type-I solution (96 spheroids/ml gel) to allow three-dimensional cell sprouting and growth. Five hundred microliters of spheroid-containing collagen gel solution was pipetted into each well of a 24-well plate and polymerized by incubation at 37°C. All cultures were maintained in EBM-2 medium, with the media changed twice a week.

### RNA isolation and polymerase chain reaction

Total RNA was extracted and purified using RNeasy Micro Kits (Qiagen GmbH, Hilden, Germany). Reverse transcription was performed using 2 µg of RNA from each sample, random primers (Promega) and M-MuLV reverse transcriptase (Peqlab). Polymerase chain reaction (PCR) was performed with Taq DNA polymerase (Life Technologies, Darmstadt, Germany) to evaluate the expression of endothelial markers on the mRNA levels using the following primers [20]: GAPDH (pos401-5′-cgtcttcaccaccatggaga, pos700-3′-cggccatcacgccacagttt), CD31 (pos234-5′-gagtcctgctgacccttctg, pos583-3′-cactccttccaccaacacct), CD34 (pos659-5′-tgaagcctagcctgtcacct pos1118-3′- gaatagctctggtggcttgc), CD146 (pos561-5′-ggccggcctctgaaggagga, pos948-3′-gcaccaggaccccgttgtcg), and vWF (pos311-5′-atgattcctgccagatttgc, pos638-3′-agactctttggtccccctgt).

### Assessment of clinical scores

To elucidate the association of injury severity and clinical parameters with the number of circulating CD34+cells, both the acute physiology and chronic health evaluation II score (APACHE II [24]) and the Sequential Organ Failure Assessment Score (SOFA [25]) were recorded and correlated with the number of circulating CD34+cells. To make the severity classification more independent of treatment, the APACHE II score was determined at admission. The SOFA score, which is usually used to track a patient's status during the course of treatment, was assessed at days 1, 3, and 7 and correlated with the respective cell numbers.

### Statistical Analysis

The primary goals of this study were to determine the number and the differentiation capacity of CD34^+^ cells in patients with severe trauma. Other cell surface markers were investigated as secondary variables. The sample size calculation was performed based on a study published by Laing *et al*. [14], which described the number of CD34^+^ cells in patients with isolated, low-energy, closed tibial fractures. A total of 16 patients (8 patients per group) were needed to obtain a desired statistical power of 0.9 (anticipated effect size (Cohen's *d*): 2.9; probability level for a two-tailed hypothesis (*p*): 0.001). All in vitro experiments were performed in a triplicate for each individual donor sample. Measurement values were expressed as the mean ± standard deviation (*SD*) of the mean or medians and quartiles/interquartile ranges (IQR), if reasonable. Data distributions were depicted in box plots. Differences between the means were compared using the non-parametric Mann-Whitney-U-Test. Correlation analyses were performed using the bivariate Pearson's correlation test. Differences between the two groups were considered to be significant at *α*≤(5%/number of comparisons), according to Bonferroni's correction for multiple comparisons. Statistical analysis was performed using the SPSS 19.0 software.

## Results

### Patient's characteristics

A total of twenty patients with severe trauma (n = 13 male, n = 7 female, mean age 46.5±17.2 years) were included in the study. An age- and gender-matched population of fourteen healthy volunteers (10 male, 4 female, mean age 43.4±19 years) was used as a control group (Table 2). There was no statistical difference in the mean age between the groups. Clinical characteristics such as age, gender, ISS score, and the number of RBC and FFP units within the first 7 days are depicted in Table 2. The median ISS Score in the ST group was 35 points (IQR 13 points). All of the patients survived the first seven days after trauma. One out of twenty patients died at day 10 after injury due to acute heart failure (female, 31 years old, ISS score 75 points).

**Table 2:** Clinical and laboratory data of the study cohort.

		ST-Group		Control Group	
		male	female	male	female
	**Age (mean; ±SD)**	45.8±20	48.0±12	41.9±20.2	47.2±17.9
	**Gender (n)**	13	7	10	4
	**ISS (points, median (IQR))**	34 (16)	37 (13)	n.a.	n.a.
	**Diabetes (n)**	0	1	1	1
	**Hypertension (n)**	3	1	1	2
	**RBC (n)**	20.9±27.8		n.a.	n.a.
	**FFP (n)**	6.6±817		n.a.	n.a.
**APACHE II (points) predicted death rate]**	**Day 1**	16.9±4.5 [27.4±12.6%]			
**SAPS-2 (points)**	**Day 1**	52.9±15.8			
**SOFA (points)**	**Day 1**	7.1±4.3			
	**Day 3**	6.0±4.5			
	**Day 7**	4.7±4.2			
**CRP (mg/l)**	**Day 1**	1.4±1.2			
	**Day 3**	194.7±93.2			
	**Day 7**	130.9±74.9			
**WBC (cells/nl)**	**Day 1**	14.4±6.9			
	**Day 3**	10.9±4.3			
	**Day 7**	9.1±2.0			
**Thrombocytes (cells/nl)**	**Day 1**	262.4±107.1			
	**Day 3**	136.5±64.7			
	**Day 7**	193.2±91.7			
**Hb (g/dl)**	**Day 1**	12.3±3.1			
	**Day 3**	10.1±2.2			
	**Day 7**	9.6±1.5			
**Hematocrit (%)**	**Day 1**	36.1±9.0			
	**Day 3**	29.2±6.5			
	**Day 7**	28.4±4.0			
**Lactate (mmol/l)**	**Day 1**	3.4±3.2			
	**Day 3**	2.4±2.3			
	**Day 7**	1.0±0.5			
**PCT (ng/ml)**	**Day 1**	n.a.			
	**Day 3**	1.4±1.1			
	**Day 7**	0.9±1.3			

RBC: red blood cell concentrate, FFP: fresh-frosen plasma, CRP: C-reactive protein, WBC: white blood cell count, Hb: hemoglobin, PCT: procalcitonin.

### The numbers of CD34^+^ and CD31^+^ cells significantly increase after severe trauma

**Figure 1:**
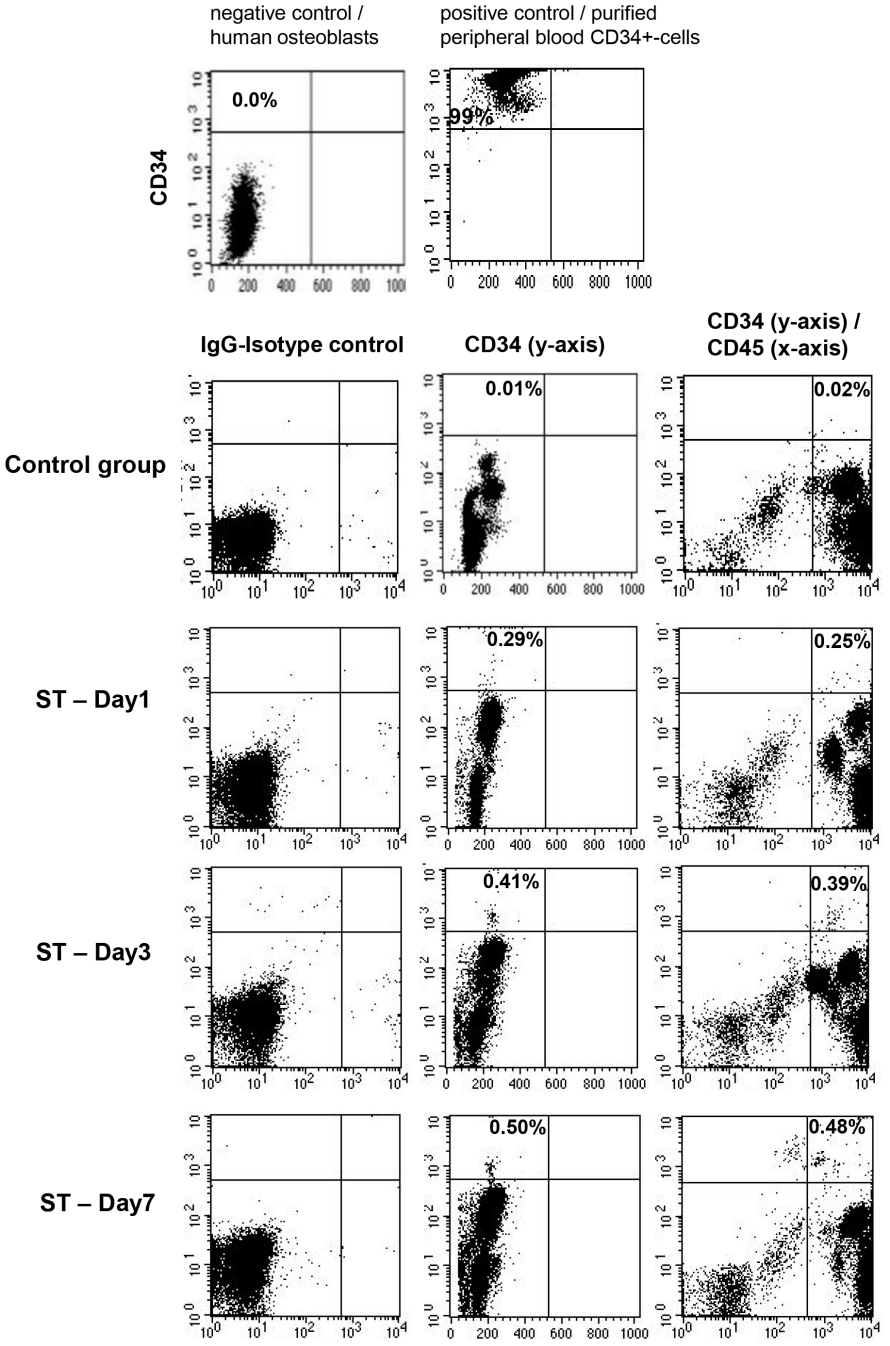
Representative examples of flow cytometric analyses for CD34 and CD45 surface antigens in a patient with severe trauma on days 1, 3, and 7 after trauma and in an age and gender-matched healthy volunteer (control group). Purified peripheral blood CD34+ cells served as positive, primary human osteoblasts as negative controls for appropriate gating of cells.

**Figure 2:**
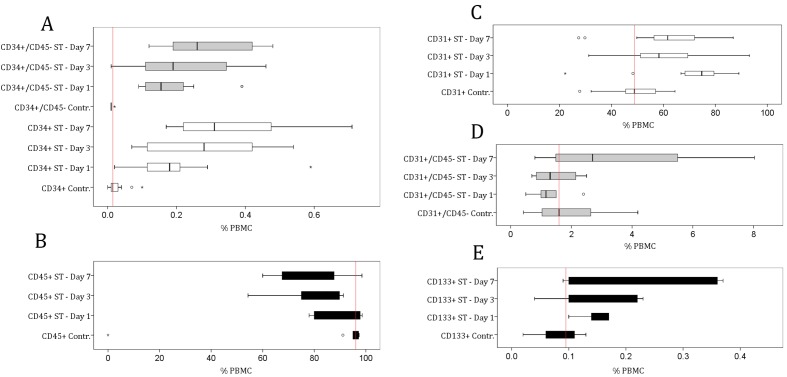
Distribution of measurement values in the control group (Contr.) and the group with severe trauma (ST) at days 1 (d1), 3 (d3), and 7 (d7). The relative numbers (%) of CD34^+^ (d1, d3, and d7), CD31 (d1 and 7), and CD34^+^/CD45^−^ cells (d 7) were significantly increased in the ST group during the period of investigation, as compared with the control group (Mann-Whitney-U-test, see Table 3 for p-values). In contrast, the number of CD45^+^ cells was significantly lower on day seven in the ST group. The total number of CD34^+^ cells (Table 3) was sufficient for isolation and further culturing procedures.

The first step in our study was to analyze the prevalence of CD34^+^ cells in the peripheral blood of severely traumatized patients (Fig. 1–2). The number of CD34+cells was quantified and compared to the control group (Table 3, Fig. 2a). Significant physiological fluctuations of cell numbers of PBMCs, CD34+, CD31+, CD45+, CD133+, CD34+/CD45-, and CD31+/CD45- were excluded in the control group at *p*-values of >0.5 in all comparisons between different time points of measurements (Mann-Whitney-U-test for data distribution at days 1 vs. 3, days 1 vs. 7, and days 3 vs. 7),

**Table 3:** Percentages of circulating cells detected by flow cytometry in PBMCs.

		ST-Group (%)	Control Group (%)	
**Day 1**	CD34	**0.18±0.13 (** ***p*** **<0.0001)**	0.03±0.03	
	CD31	**69.9±18.3 (** ***p*** ** = 0.001)**	49.2±11	
	CD45	89.5±9.5	95.8±2.1	
	CD34+/CD45-	**0.2±0.09 (** ***p*** ** = 0.001)**	0.01±0.004	
	CD31+/CD45-	1.3±0.6	1.9±1.3	
	CD133	0.14±0.03	0.08±0.04	
**Day 3**	CD34	**0.33±0.29 (** ***p*** **<0.0001)**		
	CD31	60.5±15		
	CD45	**80.6±13.4 (** ***p*** **<0.001)**		
	CD34+/CD45-	**0.22±0.15 (** ***p*** ** = 0.001)**		
	CD31+/CD45-	1.5±0.7		
	CD133	0.15±0.08		
**Day 7**	CD34	**0.37±0.2 (** ***p*** **<0.0001)**		
	CD31	**61.8±16 (** ***p*** ** = 0.007)**		
	CD45	**77.5±13.5 (** ***p*** ** = 0.006)**		
	CD34+/CD45-	**0.3±0.1 (** ***p*** ** = 0.001)**		
	CD31+/CD45-	3.6±2.8		
	CD133	0.23±0.13		

Significant differences between the experimental and the control groups are depicted in bold letters (Mann-Whitney-U-test).

We found that the number of CD34+cells in the severely traumatized patients was significantly higher at all investigated time points (6, 10 and 12-fold increases on days 1, 3, and 7; *p*<0.0001) when compared with the control group, indicating that CD34+cells are systemically recruited from the bone marrow or other niches of the body as a result of multiple injuries.

These results led us to further examine the prevalence of CD34^+^ cells that did not express the cell surface antigen CD45; therefore, they are unlikely of leukocyte origin. Interestingly, during the course of this investigation, the entire CD45^+^ population in the ST group significantly decreased in number (day 1: 89.5%; day 3: 80.6%; and day 7: 77.5% of PBMCs); however, the number of CD34^+^/CD45^−^ cells increased 200-fold on days 1 and 3 and 300-fold on day 7 after injury (*p*<0.001) in comparison to the control group (Fig. 2a, Table 3). The CD45^+^ population was significantly lower in relative number (%) in the ST group than the control group (day 3: 0.84-fold, day 7: 0.81-fold, *p*<0.001, Fig. 2b).

To address the question of whether the numbers of circulating mature endothelial cells and endothelial progenitor cells are elevated in severely injured patients, we also assessed the prevalences of CD31^+^, CD31^+^/CD45^–^, and CD133^+^ cells. The entire population of CD31^+^ cells was significantly elevated 1.42-fold and 1.26-fold on days 1 and 7 (*p*<0.01), respectively, in comparison with the control group (Table 3, Fig. 2c). The null hypothesis for temporal changes of cell numbers of CD31^+^/CD45^–^ and CD133^+^ cells in the ST group was not rejected at p-values given in Table 3 after Bonferoni's correction for multiple testing (Fig. 2d and e).

### Expression of endothelial marker molecules is significantly increased in CD34^+^-progenitor cells after severe trauma

**Figure 3:**
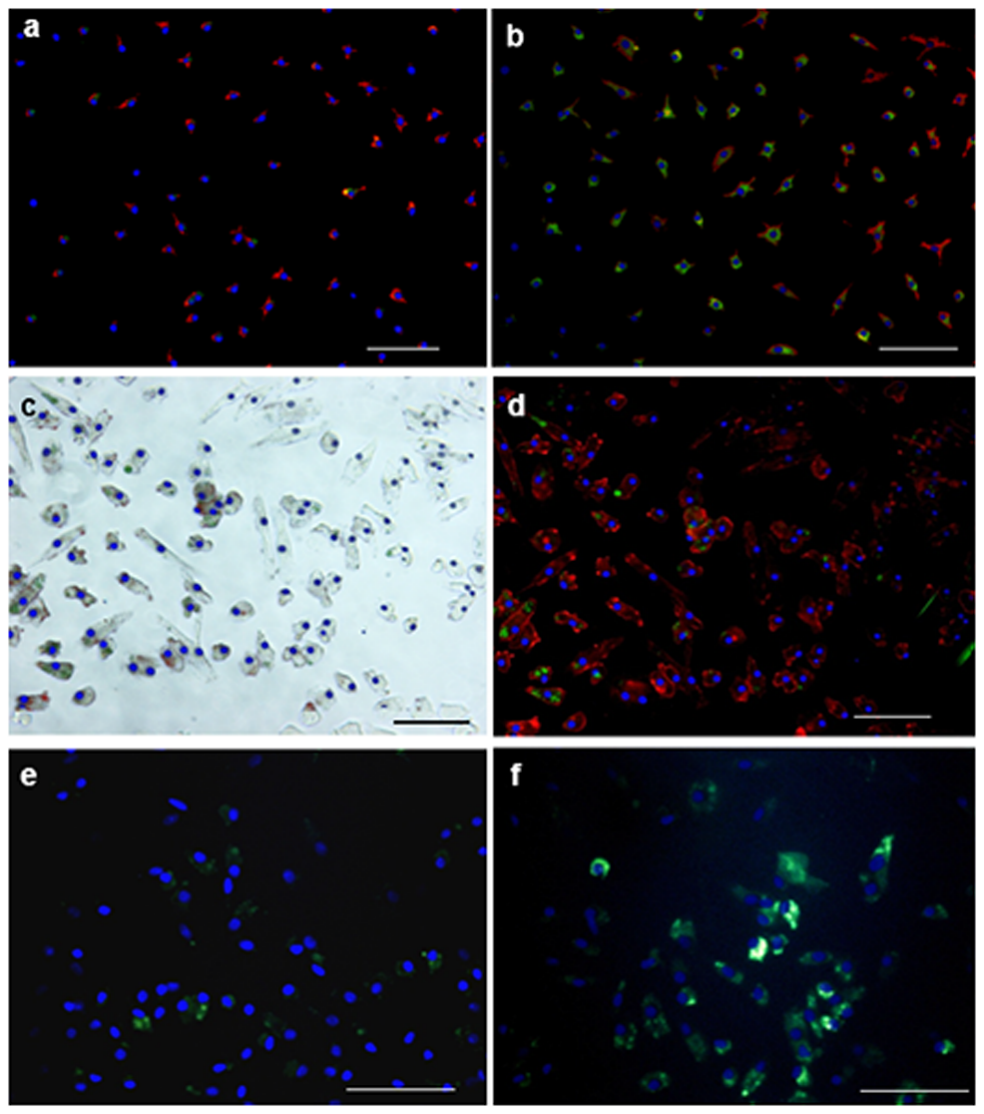
LDL-lectin and immunofluorescence staining analyses of CD34+-cells a-b: DiI-ac-LDL/lectin double staining of CD34^+^ cell cultures. **a**: control group, **b**: ST group. **c**: phase contrast/Hoechst 33258 staining of purified CD34+ST cultures (cell nuclei stained in blue), same picture as **d**: CD31 (red)/CD34 (green)/Hoechst 33258 (blue) – immunofluorescence staining showing positive reaction with cultured cells. **e-f**: CD146 (green)/Hoechst 33258 (blue) – immunofluorescence staining showing low cell numbers with positive staining in the control group (e) but positive reactions in cultured cells isolated from patients with severe trauma (f). All scale bars are 100 µm.

**Figure 4:**
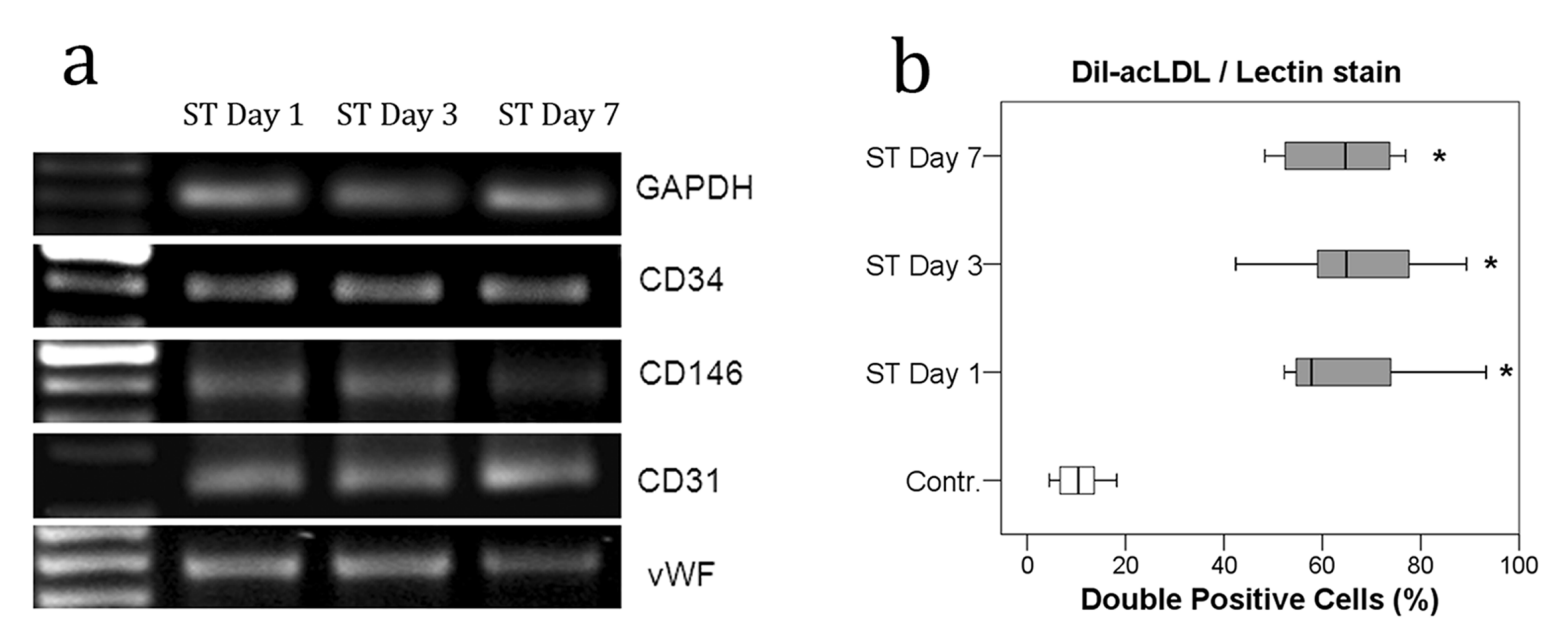
PCR analysis of endothelial marker molecules CD31, CD34, CD146, and vWF revealed positive gene expression in CD34+ cells at all time points of investigation (a) and quantitative measurement of DiI-ac-LDL/lectin double staining in CD34+ cell cultures (b); distribution of the measurement values. At all three time points, differences compared to the control group were statistically significant. *p<0.001 (Mann-Whitney-U-test).

Twenty-four hours after isolation and plating, ten percent of the cultured CD34+cells from healthy donors revealed positive double staining with DiI-ac-LDL/lectin, which is generally considered to be specific for cells of endothelial lineage (Fig. 3a and 4b). In contrast, the CD34^+^cell cultures from the severely traumatized patients contained 64 ±14% (d1, *p*<0.0001; Fig. 3b and 4b), 66±12.7% (d3, *p*<0.0001), and 63±10.3% (d7, *p*<0.0001) double positive cells, indicating that the CD34^+^ cells differentiated into the endothelial lineage in the patients with severe trauma.

**Figure 5:**
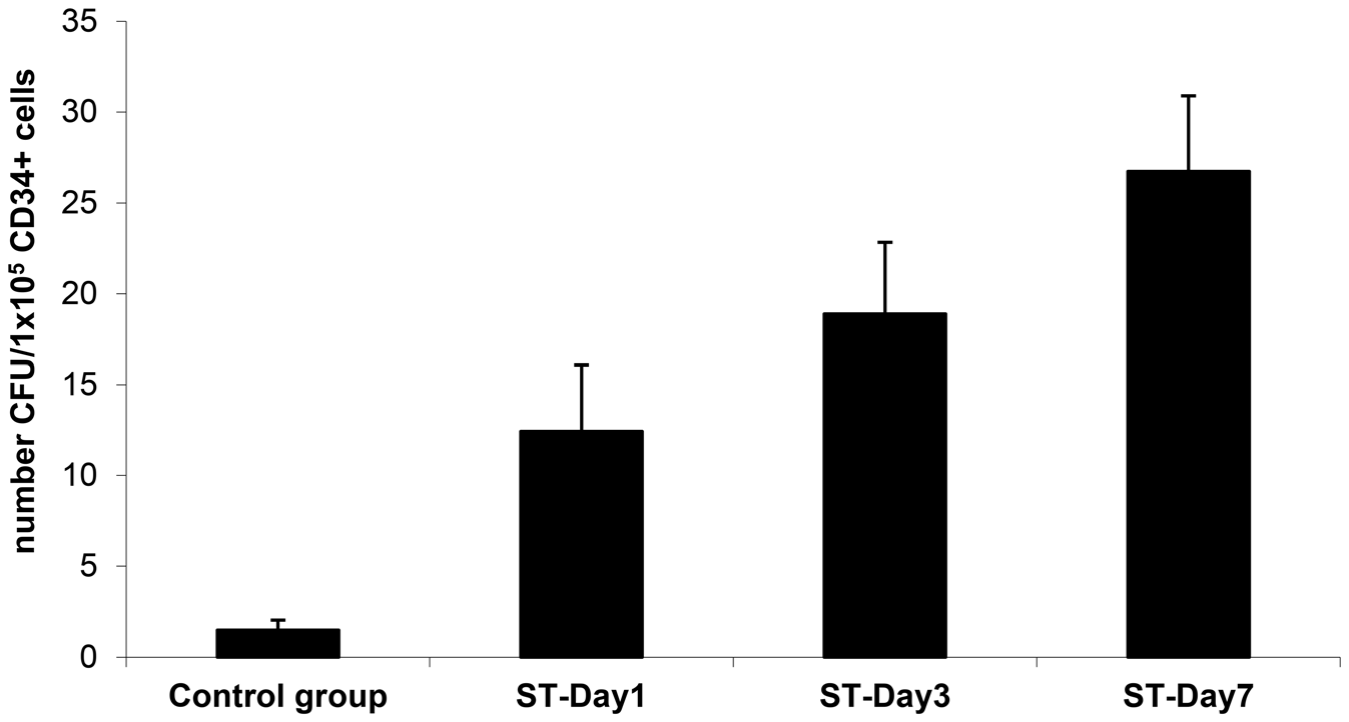
Numbers of colony forming units of endothelial cells measured five weeks after seeding 1×10^5^ of CD34+ cells purified from PBMCs of healthy volunteers (Control group) and of severe trauma patients 1, 3, and 7 days after trauma.

CD34+cells were subsequently expanded *in vitro* using standard protocols for endothelial cell culturing on fibronectin-coated surfaces. After 3–4 weeks in culture, the first colony forming units (CFUs) of elongated, spindle-like cells could be detected. In control cultures, only 1.5±0.5 CFUc were detected after a culture period of 5 weeks (Fig. 5). In clear contrast, cell cultures isolated on days 1, 3, and 7 after severe trauma generated 12±4, 19±4, and 27±4 CFUs, respectively (Fig. 5).

An analysis of endothelial cell marker expression in ST cultures by immunofluorescence revealed the positive expression of PECAM-1/CD34 and CD146 in these cells (Fig. 3c–f). Although the expression of endothelial cell marker vWF was found by PCR (Fig. 4a), we could not detect vWF expression by immunofluorescence in either group, probably due to a very low level of gene expression (not shown).

**Figure 6:**
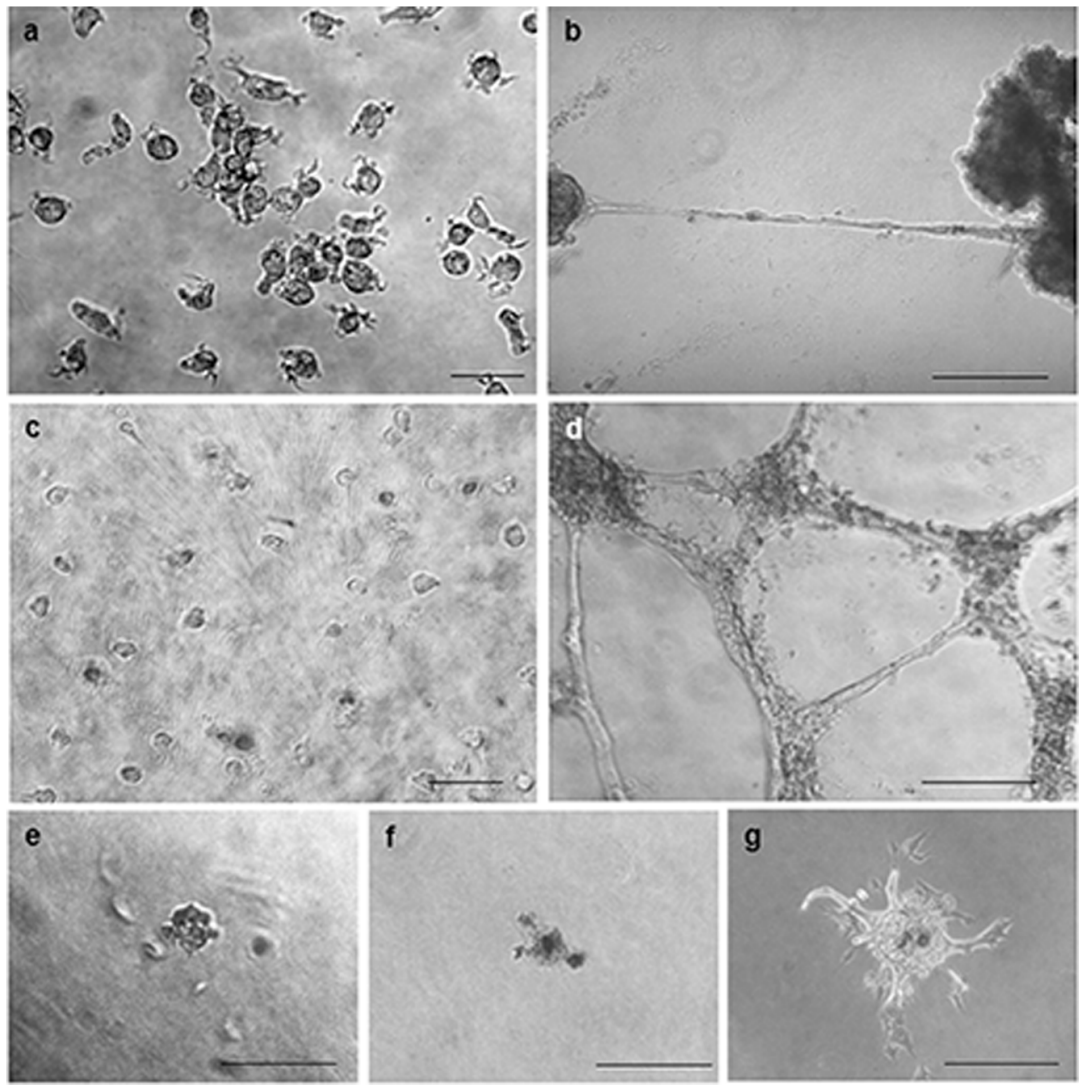
Immunohistological stainings a–d: Morphology of cells cultured on Matrigel, a: control group, b: ST group, c: CD34-depleted cell fraction (negative control), d: HUVEC cells (positive control). No formation of cord-like structures could be detected in control- and CD34-depleted cell cultures. Formation of cord-like structures was present in the ST group, but to a much lower extent as compared with HUVECs. **e–g**: Endothelial cell sprouting from carboxymethylcellulose spheroids. No formation of sprouts could be detected in the control group (**e**) whereas primitive endothelial sprouts were monitored in the ST-group (**f**). However, these formations were much less compared to HUVEC-sprout formation (**g**). All scale bars are 100 µm.

Analysis of cell morphology in Matrigel and spheroid cultures showed that CD34^+^ cultures in the ST-group contained an enriched population of elongated, spindle-shaped cells. In clear contrast to the control group, which revealed neither cord-like structures nor primitive network formations, cell cultures in the ST-group showed similar formation of cord-like structures as previously described for putative progenitors of endothelial cells (Fig. 6 a–d) [26]. However, formation of both primitive capillary structures on Matrigel and endothelial sprouting from spheroids were to a much lower extent as found in HUVEC-cultures (Fig. 6 d-g). These results indicate that CD34+cells have the potential to differentiate into the endothelial lineage *in vitro* but that they likely possess a phenotype that may be different from mature endothelial cells.

### Correlation with injury severity and patient's condition

Pearson's bivariate correlation analyses were performed to address the question of whether the number of CD34+cells was associated with the injury severity (ISS-score) and the patient's condition (assessed by the mortality rate, the APACHE-II score, and the SOFA-score) after severe trauma. We found that the number of these cells was not correlated with the ISS and the mortality rate - all but one patient survived - indicating that the amount of CD34^+^ cell recruitment was not associated with the severity of the injuries. According to these results, we also did not find any correlation with the APACHE-II score, which is highly predictive for the survival rate [24], or with the sequential organ failure assessment score (SOFA), which represents the extent of a person's organ function (Table 4).

**Table 4:** Pearson's correlation analysis.

Pearson's correlation		APACHE II (d1)	ISS (d1)	SOFA (d1)	SOFA (d3)	SOFA (d7)
**CD34+ (d1)**	***r***	0.009	0.03	0.2		
	***p*** ** (2-tailed)**	0.9	0.9	0.4		
**CD34+ (d3)**	***r***	0.6	0.22		0.3	
	***p*** ** (2-tailed)**	0.005	0.3		0.1	
**CD34+ (d7)**	***r***	0.06	0.4			−0.2
	***p*** ** (2-tailed)**	0.8	0.05			0.4

At the defined level of α, no statistically significant differences were detected.

## Discussion

CD34^+^ cells represent a heterogeneous fraction of enriched endothelial/hematopoietic progenitors in peripheral blood that give rise to endothelial cells [5], [26] and mesenchymal cells, including osteoblasts [27]. These cells have been a focus of musculoskeletal research due to their supposed therapeutic potential in wound and fracture healing [6], [11], [13]. In this study, we showed for the first time that the numbers of pro-angiogenic CD34+cells are significantly increased in severely traumatized patients during the first week after trauma. We also found that the number of circulating cells expressing CD31 (PECAM-1), a marker of mature endothelial cells, was consistently elevated in these patients. Furthermore, our *in vitro* analyses revealed significantly increased expression levels of endothelial marker molecules and endothelial cell growth in cultured CD34+cells, as shown by DiI-ac-LDL/lectin double staining, by the expression of endothelial markers (CD31, CD34, and CD146, Fig. 3 a-b and 4a), and by increased numbers of CFUs (Fig. 5). With respect to the nature of CD34+cells that do not exclusively represent the endothelial lineage, but which contain monocyte precursors as well; our results indicate that the differentiation of circulating CD34^+^ cells was clearly directed towards the endothelial lineage after severe trauma which might be due to increased numbers of endothelial progenitor cells within the fraction of CD34+ cells (e.g. CD34+/CD133+ cells).

Elements of EPC recruitment and homing are presumably promoted by the release of pro-angiogenic cytokines [28], [29]. Henrich *et al*. showed that the sera from patients with multiple traumas contain soluble factors that promote the *in vitro* differentiation of EPCs and that TGF-beta1 (see also [30]) and VEGF may be involved in their recruitment [12], [28]. Significant increases in the number of circulating EPCs have been detected in humans and animals after traumatic brain injuries [31], skin burns [32], [33], sepsis [34], [35], heart failure [36]and acute lung injury [37], [38]. Furthermore, some authors reported the mobilization of CD34+cells from bone marrow after an isolated bone fracture in rodents [10], [11] and humans [14]. As a result, the stromal cell-derived factor-1/CXC chemokine receptor 4 (SDF-1/CXCR-4) axis has been recognized as a pivotal mechanism for the recruitment of EPCs to ischemic and damaged tissues [39], [40].

A series of clinical and experimental studies, prompted by the discovery of circulating EPCs, has provided insights into these processes and rendered a new perspective for the application of new therapeutic approaches[15], [41]. Although there is increasing evidence that upon stress, sepsis, and vascular injury, EPCs are systemically mobilized in humans [14], [29], the biological function of these cells remains largely unknown. Interestingly, Matsumoto *et al.* showed that in mice, enriched fractions of EPCs were recruited to fracture sites following intravenous injection. Furthermore, the local transplantation of human CD34^+^ cells into the sites of fracture non-unions resulted in an improvement in fracture healing in nude rats. This effect may be explained by both the angiogenic and osteogenic potentials of CD34^+^ progenitor cells [6], [27]. Kuroda *et al*. reported the successful transplantation of G-CSF-mobilized, autologous CD34^+^ cells in a patient with a tibial non-union [42], which resulted in a fracture union three months after the transplantation. Although these reports strongly suggest the therapeutic potential of CD34+cells for alternative wound and fracture healing approaches, we cannot conclude from our results whether increased numbers of circulating CD34+cells are beneficial or detrimental for patients with major trauma.

With regard to their prognostic value, the association of endothelial cells with clinical outcomes has been, in part, controversially discussed for different pathologic conditions. Numerous studies reported a strong association between the levels of circulating CD34+cells with outcome parameters after cardiovascular events [2]–[4]. Liu *et al*. found a clear correlation between the levels of circulating CD34^+^/CD133^+^ cells and an improvement in the GCS scale in patients with traumatic brain injuries [31]. However, in patients with sepsis, the prognostic value of CD34+cells remains under debate [29]. In a recent study, Xin-Long *et al*. investigated the correlation between the numbers of endothelial progenitor cells expressing cell surface antigens CD34 and CD133 and the injury severity score in patients with fractures [13]. According to the results of our investigation, they found no correlation between these two parameters. In our study, the number of circulating CD34+cells did not correlate with any of the clinical outcome parameters, such as the injury severity score, survival, development of multiple organ failure, and the APACHE II scores. We suggest that the mobilization of CD34+cells and/or endothelial cells may not be a dose-dependent effect, or it may reach maximum levels in patients with major trauma.

### Study design

Focusing on the early post-traumatic phase, the patients enrolled in this study were not selected by the nature of their traumatic injury. To investigate the mobilization kinetics of CD34+cells as a general response to multiple blunt injuries, rather than on specific injury patterns, the selection was met only by the definition of severe trauma, according to well-accepted ISS score criteria [43]. The time points for the collection of blood samples were chosen according to our pilot study, which revealed that levels of CD34^+^ recruitment peaked at day 3 after trauma. To obtain a purified fraction of CD34+cells for cell culture and for an analysis of their endothelial differentiation capacity, we used a magnet-activated cell sorting technique (MACS; Miltenyi Biotec). Because the volume of blood in our population of severely injured patients was restricted, further expression analyses of subsets of cell surface antigens could not be performed in this study to better characterize the cell phenotypes. However, the results of this study strongly suggest that this number of isolated CD34^+^ cells may be sufficient for the development of cell-based therapeutic approaches, even in patients with severe trauma.

### Limitations of the study

Although we could clearly show that the number of CD34+cells and their differentiation capacity significantly differs from those in the control group of healthy individuals, conclusions cannot be drawn from this study regarding the biological function of CD34+cells. Furthermore, we cannot conclude that multiple blunt injuries are specific causes for the recruitment of CD34+cells, taking into account that these cells may be associatively mobilized with other hematopoietic cells due to a release of different cytokines. In particular, we are also aware that factors such as drugs, surgery, and nutrition may have an impact on the recruitment kinetics of these cells. The sample size of twenty consecutive patients precludes further subgroup and multivariate analyses. However, we continue to recruit patients to define the prognostic value of CD34+cells for patients with severe trauma.

Assessment of clinical scores in patients with severe trauma (APACHE II, SOFA, ISS) may be associated with serious limitations with respect to their predictive value for outcomes. The suitability of these scores and their advantages and disadvantages in comparison to other scores like TRISS, NISS etc. is still has been controversially discussed in the past. The results and conclusions of this study are therefore limited only to the assessed scores and do not describe a whole spectrum of possible relationships.

Important goals for future research are to elucidate the long-term temporal changes of CD34-PC numbers and their possible association with the outcome results and the roles of mobilization and the endothelial differentiation of CD34+cells in soft tissue and organ recovery, as well as in post-traumatic systemic insults (such as sepsis), to establish critical determinants of morbidity and mortality and to evaluate the possibilities for therapeutic gain.

## Conclusions

Our results clearly indicate that pro-angiogenic cells are systemically mobilized after severe trauma and that their numbers are sufficient for isolation and development of novel therapeutic models in regenerative medicine. However, the mobilization of these cells may not be a dose-dependent effect in patients with major trauma.
