# Correction: Mobilization of CD34^+^-Progenitor Cells in Patients with Severe Trauma

**DOI:** 10.1371/journal.pone.0106165

**Published:** 2014-08-15

**Authors:** The PLOS ONE Staff

## Notice of Republication

This article was republished on Jul 31, 2014, to replace figures that were previously unreadable. Please download this article again to view the correct version. The originally published, uncorrected article and the republished, corrected article are provided here for reference.

## Supporting Information

File S1 Originally published, uncorrected article.
(PDF)

File S2 Republished corrected article.
(PDF)
